# Dissociation of the Disilatricyclic Diallylic Dianion [(C_4_Ph_4_SiMe)_2_]^−2^ to the Silole Anion [MeSiC_4_Ph_4_]^−^ by Halide Ion Coordination or Halide Ion Nucleophilic Substitution at the Silicon Atom

**DOI:** 10.3390/molecules16108451

**Published:** 2011-10-10

**Authors:** Jang-Hwan Hong

**Affiliations:** Department of Nanopolymer Material Engineering, Pai Chai University, 155-40 Baejae-ro (Doma-Dong), Seo-Gu, Daejon, 302-735, South Korea; E-Mail: jhong@pcu.ac.kr; Tel.: +82-42-520-5755; Fax: +82-42-520-5798

**Keywords:** silole anion, silacyclopentadiene, aromaticity, dimer, tricyclic diallylic dianion

## Abstract

The reductive cleavage of the Si-Si bond in 1,1-bis(1-methyl-2,3,4,5-tetraphenyl-1-silacyclopentadiene) [(C_4_Ph_4_SiMe)_2_] (**1**) with either Li or Na in THF gives the silole anion [MeSiC_4_Ph_4_]^−^ (**2**). The head-to-tail dimerization of the silole anion **2** gives crystals of the disilatricyclic diallylic dianion [(C_4_Ph_4_SiMe)_2_]^−2^ (**3**). The derivatization of **3** (crystals) with bromoethane (gas) under reduced pressure provides [(MeSiC_4_Ph_4_Et)_2_] (**4**) quantitatively. The reverse addition of **3** in THF to trimethylsilyl chloride, hydrogen chloride, and bromoethane in THF gives 1-methyl-1-trimethylsilyl-1-silole [Me_3_SiMeSiC_4_Ph_4_] (**6**), 1-methyl-2,3,4,5-tetraphenyl-1-silacyclo-3-pentenyl-1-methyl-1-silole [C_4_Ph_4_H_2_SiMe-MeSiC_4_Ph_4_] (**7**), and 1-methyl-2,5-diethyl-2,3,4,5-tetraphenyl-1-silacyclo-3-pentenyl-1-methyl-1-silole [C_4_Ph_4_Et_2_SiMe-MeSiC_4_Ph_4_] (**8**), respectively. The reaction products unambiguously suggest that the silole anion [MeSiC_4_Ph_4_]^−^ is generated by coordination of the chloride ion at the silicon atom in **3** or by the nucleophilic substitution of either chloride or bromide ion at one of two silicon atoms in **3**. The quenching reaction of **3** dissolved in THF with water gives 1,2,3,4-tetraphenyl-2-butene, the disiloxane of 1-methyl-2,3,4,5-tetraphenyl-1-silacyclo-3-pentenyl [O(MeSiC_4_Ph_4_)_2_] (**10**) and methyl silicate.

## 1. Introduction

Since the first silacyclopentadienide anion, 5-methylbenzosilole anion, was prepared in 1958 by Gilman [1], the chemistry of group 14 metalloles has developed enormously [2,3,4,5,6,7], in particular the chemistry of silole and germole dianion [8]. Among the pioneering work reported are the first synthesis of silole dianion [9] and germole dianion [10], including synthesis and trapping reactions with halides, the NMR-characterization of the aromaticity in the silole/germole dianions [10,11,12], the X-ray crystallography of the silole/germole dianions [13,14,15,16,17,18,19,20,21,22], and their computational theoretical study [23,24]. All of the silole dianion and germole dianions are reported as highly delocalized systems, regardless of their C-substituents [8]. In contrast, there have been few reports of delocalized silole anions since NMR studies of [*t*-Bu-SiC_4_Ph_4_]^−^ were reported [25]. For example, the NMR data of the transition metal complex (η^5^-C_5_Me_5_)Ru[η^5^-(Me_3_Si)_3_SiSiC_4_Me_4_] [26] and X-ray crystallography of the transition metal complex, (η^5^-C_5_Me_5_)MCl_2_[η^5^-(Me_3_Si)_3_SiEC_4_Me_4_] (E=Si, Ge, M = Hf, Zr) [27,28], dimerization of [MeSiC_4_Ph_4_]^−^ to the disilatricyclic diallylic dianion [29], and the theoretical study of the silole anion [C_4_H_4_SiH]^−^ [30,31,32,33]. Years ago, even the heavy analogue of CpLi, 1,2-disila-3-germacyclopentadienide anion, has been reported [34]. Meanwhile there has been no additional information concerning the dimerization of the silole anion and the reactivity of this anion and its dimer. Here we report the novel generation of the silole anion [MeSiC_4_Ph_4_]^−^ from the disilatricyclic diallylic dianion [(MeSiC_4_Ph_4_)_2_]^2−^.

## 2. Results and Discussion

**Scheme 1:**
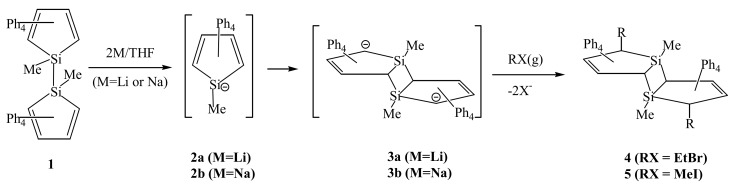
Synthesis of **3** and its derivatization with RX (EtBr, MeI).

The reductive cleavage in THF of the Si-Si bond in 1,1-bis(1-methyl-2,3,4,5-tetraphenyl-1-silacyclopentadiene) [(MeSiC_4_Ph_4_)_2_] (**1**) by either lithium or sodium gives silole anion [C_4_Ph_4_SiMe] (**2**, Scheme 1) . Upon standing at r.t. the dark blue solution of **2** gives black crystals of the respective silole anion dimer [(MeSiC_4_Ph_4_)_2_]^2−^ (**3**) whose unique disilatricyclic diallylic dianion structure has been determined by x-ray crystallography [29]. The tricyclic structure of **3** is maintained during the derivatization of **3** (crystals) with bromoethane (gas) under reduced pressure, whereupon the derivative [(MeSiC_4_Ph_4_Et)_2_] (**4**) is obtained quantitatively. In the case of iodomethane (gas) the analogous derivative [(MeSiC_4_Ph_4_Me)_2_] (**5**) is also obtained quantitatively (Scheme 1).

Surprisingly no product is obtained from the reaction of **3** (crystalline solid) with trimethylchlorosilane (gas) under reduced pressure. This can be attributed to the bulkiness of the trimethylsilyl group. It has been observed that even the smaller dimethylsilyl group exhibits hindered rotation about the bond to the ring carbon due to its steric hindrance when the dimethylsilyl group is introduced into the similar allylic anion in 1-methyl-2-dimethylsilyl-2,3,4,5-tetraphenyl-1-silacyclo-2-pentene [MeHSiC_4_Ph_4_H(SiHMe_2_)] [35].

**Scheme 2:**
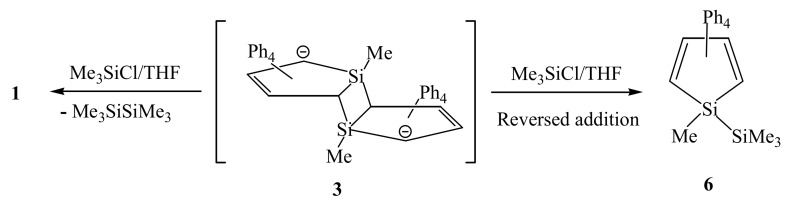
Reaction of **3** with trimethylcholorosilane.

Interestingly, the addition of the trimethylchlorosilane to **3** dissolved in THF provides only 1,1-bis(1-methylsilole) [(MeSiC_4_Ph_4_)_2_] (**1**) along with hexamethyldisilane (Scheme 2). Moreover the reverse addition of **3b** in THF to trimethylchlorosilane in THF for an extended reaction time gives [Me(Me_3_Si)SiC_4_Ph_4_] (**6**) in 65% yield (Scheme 2). These results indicate that the disilatricyclic diallylic dianion **3b** is dissociated into the silole anions **2** in THF solution. A similar dissociation of the gallole dimer [(C_4_Me_4_Ga-*t*-Bu) _2_] in benzene was previously reported [36].

**Scheme 3:**
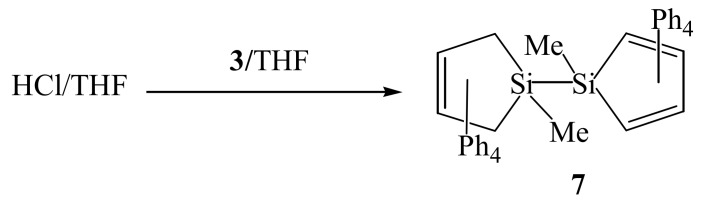
Reaction of **3** with hydrogen chloride.

Furthermore, reverse addition of **3** in THF to an excess of dry hydrogen chloride in THF affords neither a disilatricyclic derivative or the silole [Me(H)SiC_4_Ph_4_], but instead only the disilane [C_4_Ph_4_H_2_SiMe-MeSiC_4_Ph_4_] (**7**). This disilane **7** has a 1-methylsilole moiety [MeSiC_4_Ph_4_] and a 1-methyl-1-silacyclo-3-pentene moiety [MeSiC_4_Ph_4_H_2_], which is the hydrogenated derivative at the allylic 2 and 5-carboanions in the silole ring (Scheme 3).

**Scheme 4:**
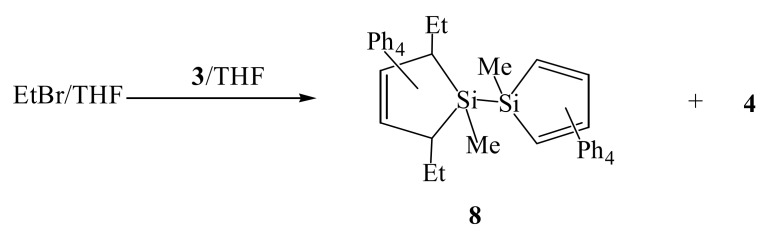
Reaction of **3** with bromoethane.

In the case of bromoethane the disilane derivative [C_4_Ph_4_Et_2_SiMe-MeSiC_4_Ph_4_] (**8**) was obtained in 65~75 % yield, along with a minor amount of the disilatricyclic derivative [(C_4_Ph_4_EtSiMe)_2_] (**4**). The disilane **8** has a 1-methylsilole moiety [MeSiC_4_Ph_4_] and the ethylation derivative at the allylic 2 and 5-carboanions in the silole ring such as **7** (Scheme 4).

**Scheme 5:**
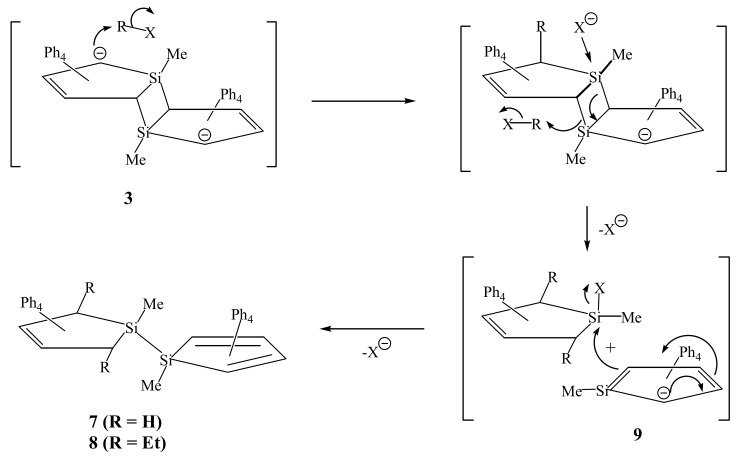
Suggested mechanism for the formation of disilanes (**7** and **8**).

The minor product **4** unambiguously indicates that some of the unique disilatricyclic structure in **3** is sustained during the reaction. However, the major products of the disilanes **7** and **8** clearly suggest that in THF the silole anion **2** and the silyl halide [R_2_Ph_4_C_4_SiMeX] (R=H, X=Cl for **7**, R=Et, X=Br for **8**) are generated in a ratio of 1:1 from **3**, as indicated in Scheme 5:

The coupling reaction of an allylic anion in one of two 5-membered rings with RX releases above the C_4_Si ring the latter’s halide ion, which is easily associated with the vicinal silicon atom of the silicon in **3**. This association easily induces ring opening in the highly strained 1-3-disilacyclobutane via a pentacoordinated state since the angles of the C-Si-C bonds in both C_4_Si ring and 1,3-silacyclobutane ring are nearly 90 degrees [29]. The nucleophilic substitution at the silicon atom produces a [Si=C] double bond in the other 5-membered ring to generate the novel silole anion [C_4_Ph_4_SiMe]^−^ (**9**) via a pentacordinated anionic intermediate, in which the methyl group and one Si-C bond of the C_4_Si ring occupy pseudo-axial positions and two bonds of X-Si-C and the other Si-C bond of the C_4_Si ring occupy pseudo-equatorial positions. Simultaneously the pushing of the electron pair in the other Si-C bond of 1,3-disilacyclobutane ring to the C_α_ carbon produces an allylic carbanion, which immediately reacts with RX. The just generated silole anion **9** is beneath the silicon atom of the C_4_Si ring, so coordination of the double bond [Si=C] to the silicon atom above of the C_4_Si ring would be an alternative mechanism involving a pseudo-pentacoordinated intermediate to stabilize the [Si=C] moiety of the silole anion **9**, such as in (η^5^-C_5_Me_5_)(PR_3_)RuH(η^2^-CH_2_=SiPh_2_) [37,38] and H_2_(PMe_3_)_3_Ru(η^2^-CH_2_=SiMe_2_) [39]. However, the preferred mechanism is a coupling reaction of the silole anion **9** with the halide. Then the nucelophilic substitution at the silicon provides the Si-Si bond of **7** or **8** while the allylic anion is rearranged to form a C=C double bond in the other C_4_Si ring to lead the silole moiety [MeSiC_4_Ph_4_].

Similar pseudo-pentacoordiated silole intermediates lacking highly electronegative atoms on the silicon atom have been proposed, and are produced by the apical attack of methyl lithium, diphenylmethylsilyl lithium [40,41], sodium bis(trimethylsilyl)amide [42], and potassium hydride [35] on the less hindered SiC_4_ ring. Even the neutral pseudo-pentacoordinate silole [Me(Me_2_NNp)SiC_4_Ph_4_] has been reported [44]. In those pentacordinated anionic intermediates it was suggested that the SiC_4_ ring occupies one axial and one equatorial position during its peudorotation and substitution reactions [45].

**Scheme 6:**
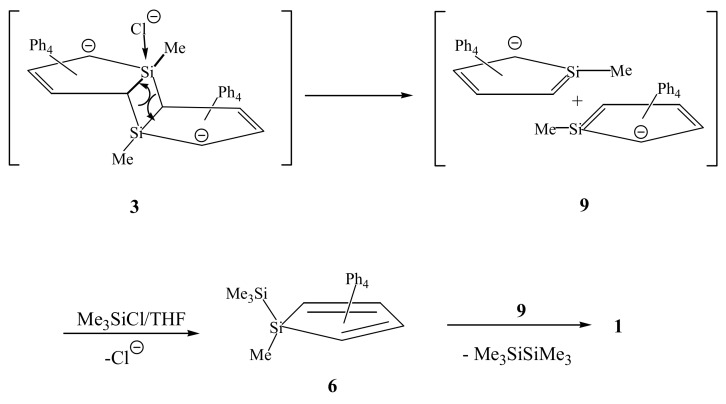
Suggested mechanism for the formation of **1**.

For some reason the reverse addition of **3** in THF to an excess of trimethylchlorosilane in THF produces chloride ion from the trimethylchlorosilane. Then, the association of the chloride ion to the silicon atom of **3** induces ring opening dissociation in the highly strained 1,3-disilacyclobutane to form [Si=C] bonds in two silole anions **9** via a pentacoordinated state, in which the methyl group and one Si-C bond of the C_4_Si ring occupy pseudo-axial positions and two bonds of X-Si-C and the other Si-C bond of the C_4_Si ring occupy pseudo-equatorial positions. Then the cation coordination enhances the stability by the delocalization in the silole ring [32]. The silole anion **9** is not consumed instantly by the reaction with trimethylchlorosilane to produce [Me(Me_3_Si)SiC_4_Ph_4_] (**6**) due to the bulkiness of trimethlsilyl group [35]. Therefore, only from the reverse addition of **3** in THF to an excess of trimethylsilylchloride for the extended reaction time **6** could be obtained (Scheme 6).

Meanwhile the weak Si-Si bond of **6** is easily attacked by the other silole anion **9** or one-electron oxidation of **9** with trimethylsilylchloride to produce only 1,1-bis(1-methylsilole) [(MeSiC_4_Ph_4_)_2_] (**1**) when trimethylsilylchloride is added to **3** in THF since the reaction rate of the silole anion **9** with trimethylchlorosilane is much slower than that of the anion **9** with the Si-Si bond of **6**. Alternatively, the silole anion **9** is persistent in the solution for a while due to the lower reactivity of the bulky trimethylsilylchloride. A similar result in the form of a radical reaction of 1,1-bis(1-phenylsilole) [(PhSiC_4_Ph_4_)_2_] has been reported by Jutzi and Karl [46].

**Scheme 7:**
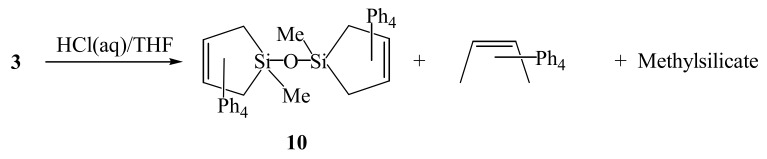
Hydrolysis reaction of **3**.

The quenching reaction of **3** dissolved in THF with water gives no 1,2,3,4-tetraphenyl-1,3-butadiene, which should be obtained if 1-methyl-2,3,4,5-tetraphenyl-1-silacyclopentadienyl moiety [MeSiC_4_Ph_4_] were present in the THF solution [47]. Instead, the disiloxane [O(MeSiC_4_Ph_4_H_2_)_2_] (**10**), 1,2,3,4-tetraphenyl-2-butene, and methylsilicate are obtained (Scheme 7).

**Scheme 8:**
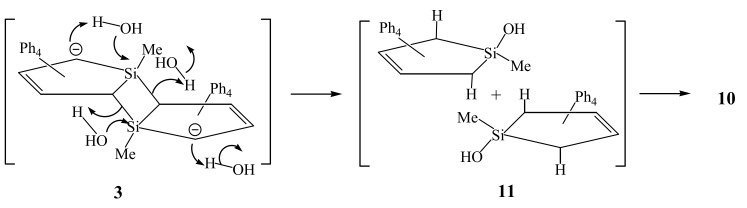
Suggested mechanism for the hydrolysis reaction of **3**.

The disiloxane **10** is the condensation product of 1-methyl-1-hydroxy-2,3,4,5-tetraphenyl-1-silacyclo-3-pentene [MeHOSiC_4_Ph_4_H_2_] (**11**), which is produced from the protonation of the allylic anions and the hydrolysis cleavages of two Si-C bonds in 1,3-disilacyclobutane ring of **3**. 1,2,3,4-Tetraphenyl-2-butene and methylsilicate are the hydrolysis products of **10** and/or **11**. These reaction products unambiguously indicate that there is no 1-methyl-2,3,4,5-tetraphenyl-1-silacyclopentadienyl moiety, but rather the disilatricyclic diallylic dianion species in the THF solution of **3** and/or the silole anion **9**. However, the self dissociation of **3** in THF to the silole anion **9** is not plausible since there is no derivatization at the silicon atom from the reaction of **3** with hydrogen chloride. Therefore it is suggested that the disilatricyclic diallylic dianion of **3** is sustained in THF solutions of **3** without halide ion to give its hydrolysis products (Scheme 8). An alternative mechanism would be involve a bent η^2^-chloride bridge between two silicon atoms of 1,3-disiacyclobutane in **3** to lead the silole anion **9** [48].

**Scheme 9:**
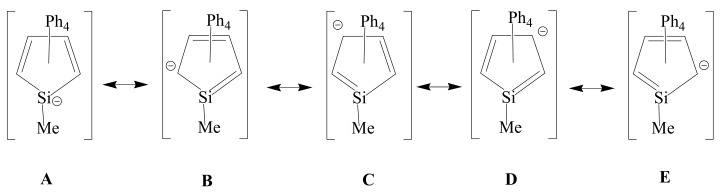
Resonance structures of the silole anion (**2**).

There are five resonance forms for the silole anion [C_4_Ph_4_SiMe]^-^ (**2**). From the reaction products the resonance form **A** (Scheme 9) can be proposed as a prominent contributor to the silole anion. The other four resonance forms are reduced to two sets of the symmetric resonance forms (**B** and **E**, **C** and **D**), which have the allyllic carbanion and silaethene moiety. The coordination of the cation, especially lithium, to the silole anion **2** enhances the delocalization in the silole ring [32], then the negative charge or electron density of the silicon atom moves to the carbons in the ring to induce some silicon-carbon double character. Consequently, the silicon atom becomes more electrophilic or less nucleophilic than that of the silyl anion **A**. It is reasoned that the the silole anion **2** has considerable Si=C double bond character and it dimerizes by head-to-tail [2 + 2] cycloaddition, which is known as silenes [49]. A *t*-Bu [25] or Si(SiMe_3_)_3_ [26] substituent on the silicon hinders the dimerization, in addition the electronic effect of the substituents on the silicon and on the carbons may be critical for the planarity of the silole anion to induce a stable 2-silaallylic anion in it (Scheme 9). It is noteworthy that the novel silole anion **9** coincides with resonance forms **B** and **D** or **E** and **C** of the silole anion **2** and it is an analogue of 2*H* or 3*H*-silole.

## 3. Experimental

### General

All reactions were performed under a nitrogen atmosphere using standard Schlenk techniques. Air sensitive reagents were transferred in a nitrogen-filled glove box. THF and diethyl ether were distilled from sodium benzophenone ketyl under nitrogen. Pentane was stirred over concentrated H_2_SO_4_ and distilled from CaH_2_. NMR spectra were recorded on Bruker WP SY and Bruker AM 200 FT-NMR spectrometers. MS data were obtained on a mass spectrometer DMX 300. IR spectra were recorded as KBR pellets on a Shimazu IR 440 and melting points were measured on a Wagner & Meunz Co. capillary type apparatus. Elemental analyses were done using a Yanaco elemental analyzer at the Analytic Center of College of Engineering, Seoul National University.

*[(MeSiC_4_Ph_4_)_2_]* (**1**): Stirring of 1-chloro-1-methyl-2,3,4,5-tetraphenyl-1-silacyclopentadiene [MeClSiC_4_Ph_4_] (4.58 g, 10 mmol) and sodium (0.23 g, 10 mmol) in THF (170 mL) at room temperature for 10 hrs produced a pale green precipitate. After evaporating THF from the mixture, the remaining solid was treated with water and dichloromethane. Removal of dichloromethane under reduced pressure from the organic suspension, gave a pale green solid. The solid was washed with ether for purification. It was identified by the comparison of its analytical data with an authentic sample [50]. Yield: 3.69 g (90%); mp 322-329 °C (lit. [50] mp > 300 °C), ^13^C-NMR (CDCl_3_, ref; ext. 77.00 ppm), −6.09 (SiMe), 154.49, 143.25, 139.76, 138.69 (C_β_, C_α_ and C_i_ of C_β_ and C_α_), 130.18, 129.34, 127.81, 127.28 (C_o_ and C_m_ of C_β_ and C_α_), 126.16, 125.52 (C_p_ of C_β_ and C_α_).

*Crystalization of* **3***:* From the dark purple THF solution, the volume of the solution reduced to a half under reduced pressure. After standing for a week it provided black crystals of **3a** and **3b,** respectively.

*[(MeSiC_4_Ph_4_Et)_2_]* (**4**): The silole anion crystals (1.42 g, 1.7 mmol for the lithium salt, 1.48 g, 1.70 mmol for the sodium salt) was exposed to ethyl bromide vapor for 2 hrs under reduced pressure. After evacuating the unreacted bromoethane, the resulting yellow solid was extracted with dichloromethane. Concentration and storage of the solution at −15 °C for a day gave yellow crystals of **4.** Yield: 1.38 g (95%) for the lithium salt, 1.38 g (95%) for the sodium salt, mp 296−300 °C; ^1^H-NMR (CDCl_3_, ref; ext. TMS = 0.00 ppm), 0.65 (s, 6H, SiMe), 0.70 (brd t, 6H, Me, J = 6.96Hz), 2.23 (brd q, 4H, CH_2_, J = 6.96Hz), 6.6-7.1 (brd m, 40H, Ph).; ^13^C-NMR (CDCI_3,_ ref; solvent = 77.00 ppm), –1.26 (SiMe_3_), 11.71 (Me), 32.48 (CH_2_), 46.33 (q-C-Ph), 43.12 (q-C-Et), 148.90, 145.01, 141.65, 141.43, 140.99, 140.62 (ethenyl C and C_i_), 132.16, 131.44, 131.09, 130.28, 127.04, 126.93, 126.62, 126.32 (C_o_ and C_m_), 126.09, 125.86, 125.28, 123.06 (C_p_).; IR (cm^−1^), δ_Si-Me_ = 1255.; MS (M^+^, relative abundance), 856 (M^+^ ,65), 797 (M^+^−59, 10), 428 (M^+^/2, 95), 135 (100).; Anal. Calcd. for C_62_H_56_Si_2_: C, 86.88; H, 6.59, Found: C, 86.86: H, 6.58.

*[(MeSiC_4_Ph_4_Me)_2_]* (**5**): Silole anion crystals (1.42 g, 1.7 mmol for the lithium salt, 1.48 g, 1.70 mmol for the sodium salt) was exposed to methyl iodide vapor for 2 hrs under reduced pressure. After evacuating the unreacted methyl iodide, the residual yellow solid was extracted with ether. Concentration and storage at −15 °C for 1 day gave yellow crystals of **4**. Yield: 0.85 g (60%) for **3a**, 1.22 g (87%) for **3b**; mp 334−338 °C (lit. [29] 336−339 °C). The NMR data of **5** agreed with those reported earlier [30].

*[Me(Me_3_Si)SiC_4_Ph_4_]* (**6**): After all volatile reagents were removed under reduced pressure, the residue was extracted with pentane. Several recrystalizations from pentane gave pure green crystals of **6** [51]. Yield; 1.15 g (65%) for the lithium salt and 1.13 g (64%) for the sodium salt, mp 130−132 °C; ^1^H-NMR (CDCl_3_, ref.: solvent = 7.27 ppm,), 0.03 (s, 9H, SiMe_3_), 0.58 (s, 3H, SiMe), 6.80-7.15 (brd m, 20H, Ph); ^13^C-NMR (CDCI_3_, ref; solvent = 77.00 ppm), −7.43 (SiMe_3_), −1.88 (SiMe), 153.84 (C_β_), 143.62(C_α_), 140.21 (Ci of C_β_), 139.26 (Ci of C_α_), 130.12, 127.27, 127.39, 127.82 (C_o_ and C_m_), 126.05, 125.36 (C_p_); ^29^Si-NMR (CDCI_3_, ref; ext. TMS=0.00), −8.13 (ring Si), −15.50 (SiMe_3_); MS (M^+^, relative abundance), 474 (M^+^+2, 21), 473 (M^+^+1. 45), 472 (M^+^, 100), 457 (M^+^−15, 42), 400 (M^+^−73, 5), 379 (48), 279(25), 221 (47), 135 (21), 105 (61), 73 (27); Anal Calcd. for C_32_H_32_Si_2_: C, 81.32 : H, 6.83, Found: C 81.52: H, 6.84.

*[C_4_Ph_4_H_2_SiMe-MeSiC_4_Ph_4_]* (**7**): Addition with stirring at room temperature of the purple solution of **3** to THF saturated with dry HCl gave a yellow solution. After removing the THF, the residue is extracted with pentane. Pentane was replaced with diethyl ether, and crystallization of the solution at −10°C for 1 day gave green crystals. Yield: 0.88g (65%) for the lithium salt, 0.85g (63%) for the sodium salt; mp 215−218 °C; ^1^H-NMR (CDCI_3_ ref.: ext. TMS = 0.00 ppm,), −0.07 (s, 3H, SiMe of butene), 0.58 (s, 3H, SiMe of butadiene), 3.53(s, 2H, CH), 6.5−7.1 (brd m, 40H, Ph); ^13^C-NMR (CDCI_3_,ref.: ext. 77.00 ppm), −7.69 (SiMe of butene), −2.71 (SiMe of butadiene), 48.41 (CH), 138.80, 140.89, 141.86, 143.01, 143.45, 155.22 (ethylene, C_i_), 129.96, 129.27, 129.12, 129.00, 128.09, 127.94, 127.52, 127.10 (C_o_ and C_m_), 126.25, 125.98, 125.44, 124.69 (C_p_); Anal Calcd for C_58_H_48_Si_2_: C,86.96; H,6.04, Found: C, 86.60; H, 6.02.

*[C_4_Ph_4_Et_2_SiMe-MeSiC_4_Ph_4_]* (**8**): Yield: 0.87 g (60%) for the lithium salt and 1.02 g (70%) for the sodium salt; mp 288−290 °C; ^1^H-NMR (CDCl_3_, ref.: solvent = 7.27 ppm,), 0.11 (s, 3H, SiMe of butene), 0.16 (s, 6H, Me, J = 6.96Hz), 0.70 (s, 3H, SiMe of butadiene), 1.86 (brd q, 4H, CH_2_), 6.6−7.2 (brd m, 40H, Ph).; ^13^C-NMR (CDCI_3_, ref.: solvent = 77.00 ppm), −4.66 (SiMe of butene), 10.56 (Me), 27.75 (CH), 49.69 (q-C), 154.85, 147.59, 144.32, 142.52, 139.52, 139.32, 139.15 (three sp^2^ C and four C_i_), 130.78, 130.61, 130.31, 129.90, 127.42, 127.35, 126.59, 126.12 (four C_o_ and four C_m_), 125.61, 125.28, 125.21 (C_p_).; IR (cm^−1^), δ_Si-Me_ = 1245; MS (M^+^, relative abundance), 856 (M^+^, 38), 457 (M^+^-Me-silole, 100), 442 (65), 427 (35), 197 (70).; Anal Calcd for C_62_H_56_Si_2_: C, 86.88; H, 6.59, Found: C, 87.20; H, 6.60.

*[(C_4_Ph_4_H_2_SiMe) _2_O]* (**10**): Aqueous HCl (0.10N) was added to a THF solution of **3** with stirring at room temperature until the pH of the mixture reached neutrality. Filtration of the hydrolyzed mixture gave a white solid, which was insoluble in organic solvents and water, did not melt when heated to over 300 °C, and showed a broad band at 1000−1100 cm^−1^in the IR spectrum. After THF was removed from the filtrate, the residue was extracted with diethyl ether. The concentrated ether solution was kept at −20 °C for 1 day and pale green crystals of **10** were obtained. The mother solution was concentrated and after standing at −20 °C for 1 day it yielded colorless crystals of 1,2,3,4,-tetraphenyl-2-butene (yield: 0.72 g, 40%), whose spectral data agreed with those reported earlier [9]. Yield(**10**): 1.35 g (33%) for **3a** and 1.23 g (30%) for **3b**; mp 292 °C; ^1^H-NMR (CDCI_3_ ref.: ext. TMS = 0.00 ppm), −0.45 (s, 6H, SiMe), 3.15 (s, 4H, CH), 6.8−7.3 (brd m, 40H, Ph); MS (M^+^, relative abundance), 818 (M^+^, 100), 650 (M^+^, 18), 537 (60), 460 (50), 445 (25), 383 (40), 382 (30); Anal Calcd for C_58_H_50_Si_2_O: C, 85.05; H, 6.16, Found: C, 84.82; H, 6.12.

## 4. Conclusions

The pathway for generation of the silole anion **9** is dependent on the reactivity of the alkyl halide used. Two silole anions **9** are generated from the disilatricyclic diallylic dianion **3** in THF by the coordination of the chloride ion to the silicon atom in it; the reaction of **9** with trimethylchlorosilane provides [(MeSiC_4_Ph_4_Me)_2_] (**5**) or [Me(Me_3_Si)SiC_4_Ph_4_] (**6**) according to the method of the addition. From the reverse addition of **3** in THF to bromoethane or hydrogen chloride in THF the silole anion **9** and the silyl halide [R_2_Ph_4_C_4_SiMeX] (R=H, X=Cl for **7**, R=Et, X=Br for **8**) are generated in a ratio of 1:1, then the coupling reaction of the two produces the corresponding disilane [C_4_Ph_4_R_2_SiMe-MeSiC_4_Ph_4_] (R=H, X=Cl for 7, R=Et, X=Br for **8**).
